# Shared Tobacco Cessation Curriculum Website for Health Professionals: Longitudinal Analysis of User and Utilization Data Over a Period of 15 Years

**DOI:** 10.2196/20704

**Published:** 2021-05-25

**Authors:** Nervana Elkhadragy, Jeremie Aviado, Henry Huang, Robin L Corelli, Karen Suchanek Hudmon

**Affiliations:** 1 School of Pharmacy University of Wyoming Laramie, WY United States; 2 Department of Clinical Pharmacy School of Pharmacy University of California San Francisco San Francisco, CA United States; 3 Department of Pharmacy Practice College of Pharmacy Purdue University West Lafayette, IN United States

**Keywords:** health professional education, interprofessional education, shared curricula, website, end-user data, tobacco cessation

## Abstract

**Background:**

Because tobacco use is a major cause of morbidity and mortality worldwide, it is essential to prepare health care providers to assist patients with quitting smoking. Created in 1999, the “Rx for Change” tobacco cessation curriculum was designed to fill an educational gap in cessation training of health professional students. In 2004, a website was launched to host teaching materials and tools to support the efforts of educators and clinicians.

**Objective:**

The objective of this study was to characterize users and utilization of a website hosting shared teaching materials over a period of 15 years.

**Methods:**

Data from the Rx for Change website have been collected prospectively since its inception. In this study, end-user data were analyzed to determine user characteristics, how they heard about the website, intended use of the materials, and numbers of logins and file downloads over time.

**Results:**

Total number of website registrants was 15,576, representing all 50 states in the United States and 94 countries. The most represented discipline was pharmacy (6393/15,505, 41.2%), and nearly half of users were students or residents. The most common source of referral to the website was a faculty member or colleague (33.4%, 2591/7758), and the purpose of enhancing personal knowledge and skills was the most commonly cited intended use of the curricular materials. A total of 259,835 file downloads occurred during the 15-year period, and the most commonly downloaded file type was ancillary handouts.

**Conclusions:**

The Rx for Change website demonstrated sustained use, providing immediate access to tobacco cessation teaching and practice tools for educators and clinicians over the first 15 years of its existence. The website has a broad interprofessional reach, and the consistent utilization over time and large number of downloads provide evidence for the feasibility and utility of a public-access website hosting teaching materials. The shared curriculum approach averts the need for educators to create their own materials for teaching tobacco cessation to students in the health professions.

## Introduction

Tobacco use is a major cause of morbidity and mortality worldwide, with more than 8 million deaths each year due to tobacco use or exposure to second-hand smoke [1]. In the United States, more than 480,000 deaths a year are attributable to cigarette smoking; of these, 33% are due to cardiovascular diseases, 27% lung cancer, 23% pulmonary diseases, 9% second-hand smoke, and 7% cancers other than lung [2]. Through multifaceted tobacco control efforts, significant progress has been made over the past several decades to reduce the overall prevalence of cigarette smoking among adults from 40% in 1964 to 14.0% in 2019 [3]. In recent years, however, the emergence of alternative nicotine delivery systems (ANDS; eg, e-cigarettes and other vaping methods) [4] has been reversing the downward trend of tobacco use, with 4.5% of adults reporting current use of e-cigarettes [3] and 20.8% currently using one or more forms of tobacco or ANDS. As such, tobacco use remains a public epidemic, predisposing individuals to an increased risk for developing diseases of virtually every organ system in the body and contributing to rising health care costs [2]. For each pack of cigarettes sold in the United States, the societal costs due to smoking-related health care costs and lost productivity are estimated at US $19.16 per pack, which is around 3 times the cost of the cigarettes [5].

It is well established that clinicians have a proven positive impact on their patients’ ability to quit [6]. To achieve reductions in the public health burden of tobacco, the 2020 Surgeon General’s report on smoking cessation highlights the importance of clinical interventions by health care providers of all disciplines [7]. Three factors should be considered when attempting to improve quit rates: (1) the efficacy of interventions on patients’ ability to quit, (2) fidelity to implementing tobacco cessation interventions in clinical settings, and (3) clinicians’ knowledge and skills for treating tobacco use and dependence. In regards to efficacy, research shows that clinician interventions that are based on the 5 A's (Ask, Advise, Assess, Assist, and Arrange) are effective and increase quit rates among patients and thus is considered a gold standard for comprehensive counseling [6]. Fidelity considers the extent to which the 5 A's are integrated into practice, in the face of challenges such as lack of time, competing demands, and lack of providers’ self-efficacy for tobacco cessation counseling [8]. To mitigate these challenges, investigators have explored creative approaches to enhance the delivery of care (eg, Satterfield and colleagues [9] found that a computer-facilitated 5 A's approach performs better than usual care). The third aspect that can influence quit rates is clinicians’ knowledge and skills for providing tobacco cessation interventions. To address this, the “Rx for Change Clinician-Assisted Tobacco Cessation” curriculum was designed, and its corresponding website [10] was launched to host the tobacco cessation teaching and counseling materials. The Rx for Change curriculum, and the website described here, aim to enhance the quality and quantity of tobacco counseling that occurs in clinical practice.

Historically, the extent of tobacco cessation content has been inadequate in all health professional school curricula, including medical [11-15], nursing [16-19], pharmacy [20,21], dental hygiene [22], physical therapy [23], physician assistant [24], and respiratory therapy [25,26]. The evidence-based Rx for Change curriculum was a practical solution to address this decades-long gap [27]. The term “Rx” means prescription, and a “curriculum” is defined as “the totality of student experiences that occur in the educational process” [28]. As such, Rx for Change is a curriculum about tobacco cessation that was designed to teach health professional students and licensed clinicians. Rogers’ Diffusion of Innovations Theory [29] served as a guiding framework for program design, aiming to enhance the adoptability of the curricular innovation and structure future dissemination strategies. A key strategy for dissemination of Rx for Change occurred via targeted in-person and virtual train-the-trainer workshops for faculty at health professional schools (pharmacy, nursing, medicine, and respiratory care).

**Figure 1:**
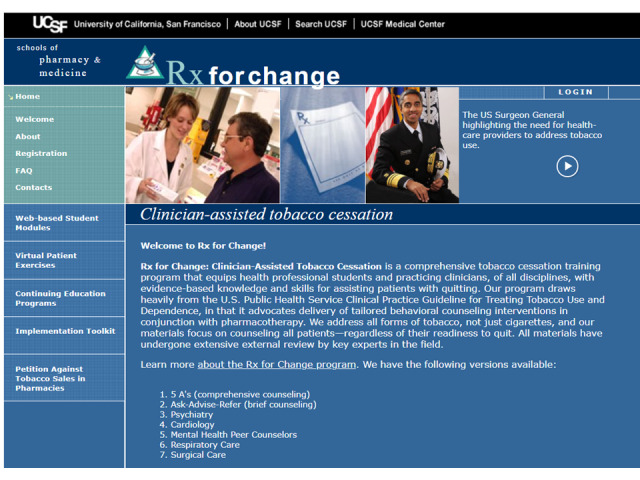
Rx for Change website homepage [10].

**Figure 2:**
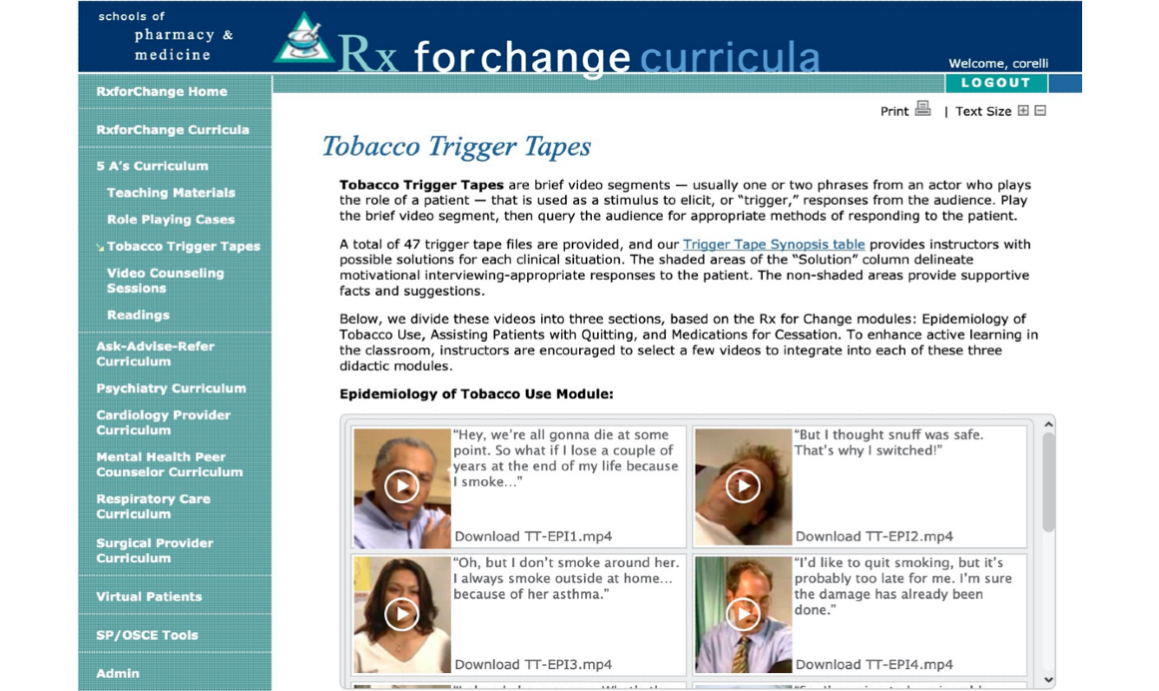
Rx for Change website: sample page hosting tobacco “trigger tape” videos.

To facilitate integration of the Rx for Change curriculum at health professional schools, a public-access website was created to host all of the Rx for Change curricular materials (Figure 1). Several versions of the curriculum exist, each addressing a different clinical specialty for which patients can benefit from tobacco cessation interventions. Learning objectives are provided for each of the program’s modules. PowerPoint slides, with detailed instructor notes, and learner handouts are downloadable and can be used by educators to teach in a lecture-based format. Additional teaching materials include dozens of videos (Figure 2), case materials for role playing, ancillary handouts for clinicians and patients, and a suite of tobacco-specific virtual patients. To facilitate assessment of counseling competencies, 6 standardized patient cases were created with associated scoring rubrics for conducting objective structured clinical examinations (OSCEs). Tools are also available to assist faculty with implementation of all aspects of the curriculum. The U.S. Surgeon General provides a 3-minute introductory video, highlighting the importance of integrating tobacco cessation into clinical practice (Figure 1).

Educational experts have placed much value on developing effective training programs and have also emphasized the need for program evaluation [30]. Unfortunately, when websites are created to host educational materials, these resources are often short-lived before becoming outdated and dormant after institutional support or grant funds expire. Launched in 2004, the Rx for Change website teaching content is updated at least annually and also when needed to address changes in clinical practice (eg, postlaunch of a new medication, inclusion or removal of a boxed warning). However, its usage has yet to be characterized. Such knowledge would be helpful to understand the impact of providing shared curricular materials through a public access website and to inform future curriculum developers about potential usage and benefits of hosting shared materials online. Therefore, the purpose of this study was to conduct a longitudinal analysis of user characteristics and utilization of the Rx for Change website over a period of 15 years.

## Methods

User and utilization data have been collected prospectively via the Rx for Change website since its launch in 2004. For the purpose of this study, data were extracted for a period of 15 years, ranging from the public launch date on April 1, 2004 to March 31, 2019. Individuals who registered on the website provided contact information, including their state and country, their primary discipline (medicine, nursing, pharmacy, respiratory care, dentistry, health educator/peer counselor, social work, other), and whether they were a student or resident. Additional information included how they heard about the Rx for Change program (conference/meeting/workshop; faculty member/colleague; internet LISTSERV; newsletter or publication; surfing the internet; University of California San Francisco Smoking Cessation Leadership Center; other) and their intended use of the materials (enhance own knowledge/skills; teach health professional students; teach licensed health professionals; not sure). In addition to user characteristics, prospectively collected data included various utilization measures: files downloaded (frequency and type), number of file downloads per user, number of logins, and trends in utilization over time. All video files on the website are permitted to be streamed directly on the website, and these occurrences were not linkable to individual users and therefore were not captured along with the number of file downloads.

With respect to data interpretation, it is important to note that not all programmatic materials were available at the launch of the website in 2004 — a version addressing brief counseling (Ask-Advise-Refer) was launched in November 2007, and new discipline-specific versions (eg, psychiatry, respiratory care, peer counselor, cardiology, and surgical care) became available over time. Along with the annual updates, new videos and role-playing case materials were added periodically, and all were modified as needed to be consistent with evolving clinical practice guidelines. In 2019, a suite of 6 standardized patient cases with scoring rubrics for OSCEs were added along with a link to a suite of tobacco-specific virtual patients [31]. No proactive efforts were made (eg, no email notifications) to alert users of the availability of new or updated content, and at no time during the 15-year period was the website inaccessible for more than a few hours at a time during updates or server maintenance.

Data cleaning occurred at the individual user level, which included combining duplicate registrants (eg, identical users who established separate accounts with different email addresses), reclassifying disciplines where appropriate, and recategorizing data response options labeled as “other” (eg, user checked “other” for the discipline field but provided information consistent with existing response options). Combining duplicate registrants was done by manually reviewing registrations that appeared to belong to the same person, and after extensive investigation through internet search engines and LinkedIn profiles, discussion, and consensus, the team determined when it was appropriate to attribute multiple registrants to the same user. Data were analyzed using SPSS, version 26 [32]. The study was approved by the University of California, San Francisco and Purdue University Institutional Review Boards for the protection of human subjects.

## Results

### User Characteristics

A total of 15,576 unique users registered on the Rx for Change website during the study period. Registrants represented all 50 states in the United States and 94 different countries. Among users with a designated health discipline (15,505/15,576, 99.5%), the top represented disciplines were pharmacy (6393/15,505, 41.2%), followed by nursing (3377/15,505, 21.8%) and health educators/peer counselors (1653/15,505, 10.7%; Table 1). Students and residents represented 49.7% (7747/15,576) of all registrants.

**Table 1:** Represented disciplines among 15,505^a^ end users reporting discipline and student or resident status.

Disciplines	Nonstudent or nonresident (n=7758), n (%)	Student or resident (n=7747), n (%)	Total (n=15,505), n (%)
Pharmacy	1790 (23.1)	4603 (59.4)	6393 (41.2)
Nursing	1305 (16.8)	2072 (26.7)	3377 (21.8)
Health educator or peer counselor	1461 (18.8)	192 (2.5)	1653 (10.7)
Medicine^b^	677 (8.7)	239 (3.1)	916 (5.9)
Respiratory care	440 (5.7)	127 (1.6)	567 (3.7)
Dentistry	174 (2.2)	87 (1.1)	261 (1.7)
Social work	112 (1.4)	21 (0.3)	133 (0.9)
Other	1799 (23.2)	406 (5.3)	2205 (14.2)

^a^71 (0.5%) end users did not provide data describing their student/resident status and discipline.

^b^Includes physicians and physician assistants.

Of nonstudents/nonresidents, approximately one third (2591/7758, 33.4%) reported hearing about the website from a faculty member or colleague; the remainder heard about the website at a conference, meeting, or workshop (1305/7758, 16.8%); while surfing the internet (1295/7758, 16.7%); on an internet LISTSERV (734/7758, 9.5%), distributed by the University of California Smoking Cessation Leadership Center (531/7758, 6.8%), or in a newsletter publication or article (468/7758, 6.0%). The most commonly selected intended use of the Rx for Change materials was to enhance personal knowledge and skills (5792/7308, 79.3%); 39.2% (2867/7308) intended to teach licensed health professionals, and 33.2% (2425/7308) indicated that they intended to teach health professional students (categories not mutually exclusive).

### Website Utilization Characteristics

**Figure 3:**
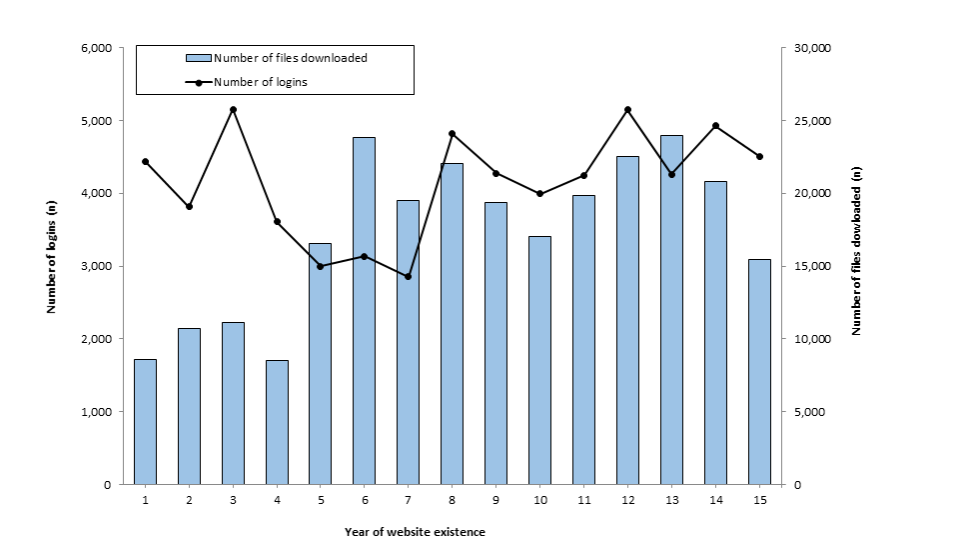
Number of files downloaded and number of logins, per year (April 1, 2004 to March 31, 2019).

During the evaluation period, 259,835 files were downloaded by 12,387 users, representing 79.5% (12,387/15,576) of all website registrants. While the remainder of the registrants (3189/15,576; 20.5%) might have streamed videos on the website, they did not download any files. The file type most commonly downloaded was ancillary handouts (n=61,348), followed by counseling videos (n=58,109) and instructors’ PowerPoint slides (n=49,501; Table 2). Across the 15-year time period, users logged into the website a total of 62,172 times. Login frequency and download frequency trends over time are shown in Figure 3.

**Table 2:** File downloads (n=259,835) by teaching tool.

Teaching tool	Description of tool	Number of downloads, n (%)
Ancillary handouts	Tools that clinicians can use when helping patients (eg, tobacco cessation counseling guide, withdrawal symptoms information sheet, drug interactions with tobacco smoke table, tobacco use log, coping strategies for patients, pharmacologic product guide)	61,348 (23.6)
Counseling videos	Video segments demonstrating counseling of a wide range of patients (not ready to quit, ready to quit, recent quitter, former tobacco user) in many patient care settings	58,109 (22.4)
PowerPoint teaching slides	PowerPoint slides with detailed instructor notes and relevant literature citations	49,501 (19.1)
Learner slide handouts	PDF versions of the PowerPoint slides	32,024 (12.3)
Role playing cases	Handouts for role playing with a wide range of patient case scenarios (not ready to quit, ready to quit, recent quitter, former tobacco user)	22,809 (8.8)
Trigger tape videos	Brief video segments (1-2 phrases from an actor who plays the role of a patient) that are used as a stimulus to elicit, or “trigger,” discussion with learners	17,959 (6.9)
Instructor tools	Guides and other resources to facilitate implementation of the Rx for Change curriculum	8749 (3.4)
Introductory videos	A 3-minute video created by the U.S. Surgeon General highlighting the need for health care providers to address tobacco use and an 8-minute introductory video of interviews with smokers	3582 (1.4)
Reading materials	Recommended background readings (eg, PDF versions of textbook chapters and continuing education programs on tobacco cessation)	3451 (1.3)
Administrative tools	End-user license agreement, sample medication order forms, tracking forms, etc.	2213 (0.9)
OSCE^a^ case materials	Standardized patient cases, with corresponding scoring rubrics for formative and evaluative exercises	90 (<0.01)

^a^OSCE: objective structured clinical examination; these competency assessment tools became available on the website in 2018.

## Discussion

### Principal Findings

This study contributes important knowledge to the literature regarding the extent to which health professional educators, clinicians, and students utilize a website that was designed to house and disseminate educational materials for tobacco cessation. The study complements our concurrent research evaluating the Rx for Change program, thus providing a more complete picture of the program’s reach and long-term impact [33]. Although an abundance of existing literature describes web-based interventions for tobacco cessation [34], to our knowledge, there are no studies that characterize internet-based access to tools designed to facilitate faculty and students in their teaching and learning roles and clinicians in their patient care roles. Current literature addressing professional educational websites other than tobacco cessation is also scarce. We identified 3 websites that house teaching materials (pharmacogenomics, infectious diseases, and diabetes mellitus) [35-37], but utilization of these sites have not been described in the literature. In addition to widespread use of the website over a period of 15 years, the Rx for Change materials have been used in a variety of tobacco cessation studies across several health disciplines [38-51]. Recently, the long-term impact of the train-the-trainer programs on faculty development and Rx for Change implementation in pharmacy schools was evaluated through application of the RE-AIM framework [52,53].

Rogers’ Diffusion of Innovation Theory [29], which was used to develop and disseminate the Rx for Change curriculum, was also used to guide elements of data interpretation. This theory states that new programs are more likely to exhibit enhanced adoption if they possess 5 main characteristics: (1) relative advantage over existing programs; (2) compatibility with existing values, experiences, and needs of potential adopters; (3) how complex the program is to understand and use; (4) trialability, or the extent to which a potential user can test or experiment with a program before committing to adoption; and (5) observability (ie, the extent to which the program provides tangible outcomes). Most users learned about the Rx for Change website from another colleague, which suggests that colleagues perceived the website and its materials to possess a relative advantage over other available sources. This perception is consistent with findings from a prior study, in which the majority of faculty respondents (89.9%) rated the website as either very or extremely useful [53]. Compatibility was shown by the fact that website registrants’ most commonly cited intention for use of the curricular materials was to enhance their own knowledge and skills. Trialability and perceived acceptability of the complexity of the Rx for Change program were evident by the large number of registrations and continued use over time. An observable result was the large number of logins and file downloads from the website.

Previous findings suggest that the availability of a website to host shared teaching materials is a useful resource for health professional educators, and users report appreciation for access to regularly updated teaching content [33]. In our study, the most frequent referral source was a faculty member or a colleague (33.4%). These findings are consistent with those identified in the evaluation of a web-based mental health portal, for which the highest utilization was among individuals personally invited to visit the website [54]. Thus, an effective mode of dissemination is learning about the program or its website from a professional or social network. Although no proactive efforts were made to alert users about updates or new content, this is a strategy that could be considered in the future as well as a brief survey of user needs to provide guidance for future program enhancements. Another area of future research is assessing important aspects of the website such as the website’s readability, quality of information, credibility, and design.

### Limitations

Limitations of this study include a possibility of duplicate users who utilized different email addresses when registering on the website. This was addressed through a manual review, as described in the Methods. Additionally, the number of file downloads found in this study is an underestimate, because videos can be streamed and viewed directly on the website, without downloading. Also, the number of file downloads likely underestimates actual utilization in the classroom or in clinical practice. For example, an instructor or clinician might download the content once and use it on a regular basis until the next update of the program materials, and these implementation activities are not captured by the Rx for Change website. This study does not provide evidence that a shared curriculum website would contribute to changes in the prevalence of tobacco use, although it is well-documented that clinicians have a proven, positive impact on their patients’ ability to quit and therefore training is warranted [6]. Finally, because the ability to evaluate the long-term utilization of the shared curricular resources is fully dependent on the ability to maintain the quality and accessibility of the materials, the sustainability of any program is significantly challenged without ongoing funding and personal commitment of the program creators.

### Conclusions

The Rx for Change website utilization data demonstrated sustained use, providing immediate access to shared, evidence-based tobacco cessation teaching and practice tools for educators and clinicians since 2004. The website had a broad interprofessional reach, which increases the likelihood of tobacco users receiving assistance from multiple types of health care providers. The consistent utilization over time and large number of downloads provided evidence for the feasibility and utility of a public access website hosting a shared tobacco cessation curriculum for health professionals. The shared curriculum concept, in tandem with a frequently updated website to host curricular materials, can be replicated for other topics of public health importance.

## Abbreviations

ANDS: alternative nicotine delivery systems
OSCE: objective structured clinical examination
